# Cytoskeletal influences on nuclear shape in granulocytic HL-60 cells

**DOI:** 10.1186/1471-2121-5-30

**Published:** 2004-08-19

**Authors:** Ada L Olins, Donald E Olins

**Affiliations:** 1 Department of Biology, Bowdoin College, Brunswick, Maine 04011, USA

## Abstract

**Background:**

During granulopoiesis in the bone marrow, the nucleus differentiates from ovoid to lobulated shape. Addition of retinoic acid (RA) to leukemic HL-60 cells induces development of lobulated nuclei, furnishing a convenient model system for nuclear differentiation during granulopoiesis. Previous studies from our laboratory have implicated nuclear envelope composition as playing important roles in nuclear shape changes. Specifically noted were: 1) a paucity of lamins A/C and B1 in the undifferentiated and RA treated cell forms; 2) an elevation of lamin B receptor (LBR) during induced granulopoiesis.

**Results:**

The present study demonstrates that perturbation of cytoskeletal elements influences nuclear differentiation of HL-60 cells. Because of cytotoxicity from prolonged exposure to cytoskeleton-modifying drugs, most studies were performed with a Bcl-2 overexpressing HL-60 subline. We have found that: 1) nocodazole prevents RA induction of lobulation; 2) taxol induces lobulation and micronuclear formation, even in the absence of RA; 3) cytochalasin D does not inhibit RA induced nuclear lobulation, and prolonged exposure induces nuclear shape changes in the absence of RA.

**Conclusions:**

The present results, in the context of earlier data and models, suggest a mechanism for granulocytic nuclear lobulation. Our current hypothesis is that the nuclear shape change involves factors that increase the flexibility of the nuclear envelope (reduced lamin content), augment connections to the underlying heterochromatin (increased levels of LBR) and promote distortions imposed by the cytoskeleton (microtubule motors creating tension in the nuclear envelope).

## Background

Granulopoiesis, the differentiation of peripheral blood granulocytes, involves dramatic nuclear and cytoplasmic structural changes [1]. Committed bone marrow progenitor cells possess ovoid-shaped nuclei with prominent nucleoli and a paucity of heterochromatin. The mature terminally differentiated human neutrophil (polymorphonuclear granulocyte) exhibits a distinctly lobulated (segmented) nucleus with shrunken nucleoli and extensive peripheral heterochromatin. The mature neutrophil is released into the bloodstream, where it circulates as a round unpolarized cell. Responding to chemotactic agents produced by infection and tissue damage, the circulating neutrophil changes cell shape, converting to a rapidly migrating polarized cell. Mature granulocytes have a limited lifespan, succumbing to apoptosis within a few days following release into the bloodstream.

Several established tissue culture cell lines have been investigated as model systems for understanding the events and mechanisms of granulopoiesis [2]. Previous studies from our laboratory have employed the HL-60 cell system to examine nuclear lobulation and cytoskeletal polarization [3-5]. HL-60 cells exhibit granulocytic differentiation in response to added retinoic acid (RA) [6], eventually undergoing apoptotic death [7]. Our studies on RA induced granulocytic differentiation of HL-60 cells implicated two major factors in the nuclear lobulation process: 1) very low cellular levels of lamins A/C and B1; 2) a significant increase in cellular levels of lamin B receptor (LBR). Ultrastructural studies suggested that during nuclear differentiation, nuclear envelope surface area was increased [3]. In addition to nuclear lobulation, these studies demonstrated the formation of extensive nuclear envelope outgrowths, denoted "nuclear envelope-limited chromatin sheets" or ELCS. The suspected role of LBR was confirmed in subsequent studies [8,9], which demonstrated that a genetic deficiency of LBR correlates with hypolobulated granulocyte nuclei in the human Pelger-Huet anomaly [8] and the murine Ichthyosis mutation [9]. LBR is an integral membrane protein of the nuclear envelope inner membrane, with putative interactions to lamin B, chromatin and HP1α [10]. The mechanistic relationships between changes in nuclear envelope composition and granulocytic nuclear lobulation are currently unknown.

The position of the interphase nucleus within the cell appears to be regulated by microtubules and associated dynein [11] and actin and associated spectrin-like proteins [12-15]. However, only fragmentary data exists examining the relationship, if any, between cytoskeletal elements and nuclear shape. Absence of intermediate filaments (vimentin) has been correlated with nuclear envelope folds or invaginations [16]. Employing HL-60 cells, evidence has been published that neither microtubules (MTs) nor the actin microfilament system are essential for the establishment of nuclear lobulation [17]. Our laboratory chose to investigate these conclusions concerning HL-60 cells in greater detail. Employing the same cell subline (HL-60/S4), we demonstrated that brief (i.e., 2 and 4 hour) treatments of undifferentiated or granulocytic (RA treated) cells with various cytoskeletal modifying chemicals had no obvious effects upon nuclear shape, although cell shape was strongly affected [4]. However, prolonged (i.e., 2 day) exposure of undifferentiated HL-60/S4 cells to nocodazole (NC, disrupts MTs) or taxol (TX, stabilizes MTs), but not cytochalasin D (CD, disrupts actin microfilaments), resulted in rapid apoptotic death. For this reason, the present study emphasizes the prolonged exposure of cytoskeleton-modifying chemicals on Bcl-2 overexpressing HL-60 cells [18,19]. These cells are more refractory to undergoing apoptosis, but can still be induced with RA to exhibit granulocytic differentiation. Employing HL-60-*bcl*-2 cells [18], we demonstrate that the integrity of the MTs system, but not the actin microfilament system, is essential for the nuclear lobulation process during *in vitro *granulopoiesis. Prolonged exposure of HL-60-*bcl*-2 cells to CD or TX does lead to perturbations of nuclear shape, independently of RA induced nuclear differentiation.

## Results

### HL-60/S4 cells undergo retinoic acid induced nuclear lobulation in the presence of cytochalasin D, but do not survive nocodazole or taxol treatment

**Figure 1:**
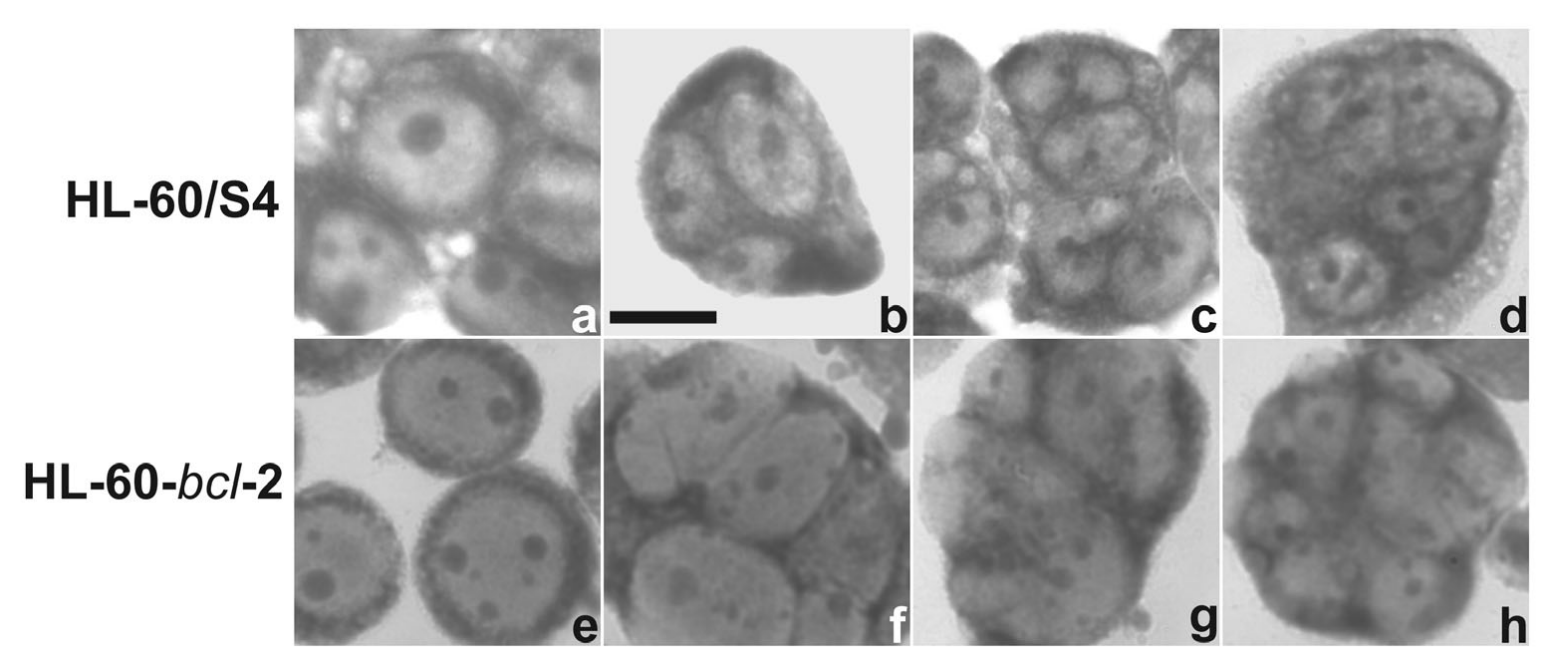
**Wright-Giemsa stained HL-60 cells following exposure to RA and CD. **HL-60/S4 cells (Panels a-d): a, undifferentiated; b, 1 μM CD; c, 1 μM RA; d, 1 μM RA plus 1 μM CD. Three days of drug exposure. HL-60-*bcl*-2 cells (Panels e-h): e, undifferentiated; f, 1 μM CD; g and h, 1 μM RA plus 1 μM CD. Seven days of drug exposure. Scale bar: 10 μm.

HL-60/S4 cells were cultivated for up to four days in medium with (or without) 1 μM RA and with (or without) 1 μM CD. Daily samples of these four cultivation conditions were cytospun on microscope slides and Wright-Giemsa stained for examination. The image data from day 3 are presented (Figure 1a,1b,1c,1d). Control cells (Figure 1a; no RA or CD) possessed single ovoid nuclei (>99% of cells) throughout the entire period; CD treatment alone (Figure 1b) exhibited primarily multinucleated cells. RA treated controls (Figure 1c; no CD) revealed clear nuclear indentation and lobulation by day 3 [3]; RA plus CD treated cells revealed indented and lobulated multiple nuclei (Figure 1d). HL-60/S4 cells exposed to 1 μM CD, with or without RA, became mostly multinucleated by day 3 (approximately 4% mono-, 56% bi-, 20% tri-, and 18% tetranucleated). Evidence of apoptosis became apparent by day 4. In addition, immunostaining of RA plus CD treated cells (day 4) with anti-lamin B (data not shown) clearly indicated stained "patches" between nuclear lobes, interpreted as ELCS [3]. These results are in complete agreement with a previous publication [17], and monitored HL-60/S4 nuclear differentiation for a longer period.

The previous publication [17] also examined the role of MTs on nuclear lobulation in HL-60/S4 cells. They exposed RA treated cells to 0.1 μM (0.03 μg/ml) NC for up to two days, concluding that the integrity of MTs was not important to the formation granulocytic nuclear lobes. We repeated this experiment, including a separate comparison of HL-60/S4 cells exposed to 0.1 μM TX. The results clearly indicated ~100% apoptotic cell death by day 2 with either NC or TX, preventing any firm conclusion of whether MT integrity is important for nuclear lobulation. We concluded that exploration of this issue would be better accomplished using HL-60-*bcl*-2 cells, which are more refractory to undergoing rapid apoptosis.

### HL-60-*bcl*-2 cells exhibit nuclear lobulation in response to retinoic acid

**Figure 2:**
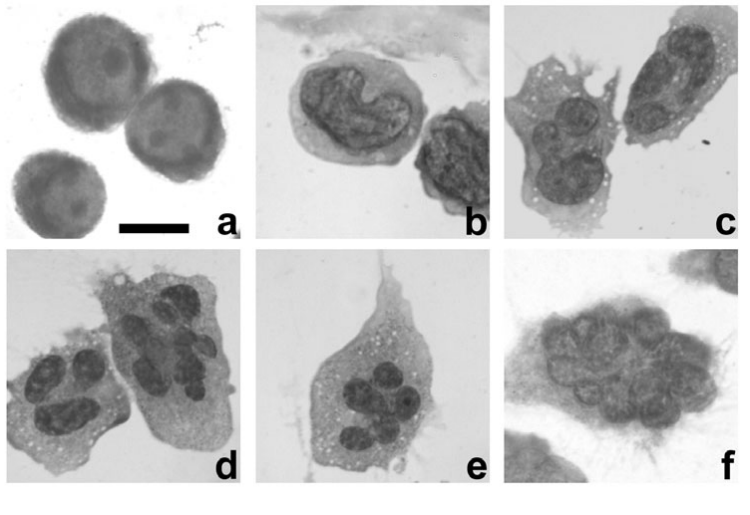
**Nuclear differentiation during RA induced granulopoiesis of HL-60-*bcl*-2 cells monitored by Wright-Giemsa staining. **Examples of the four nuclear morphological states: a, ovoid; b, indented; c, lobulated; d, lobulated (left) and multilobed (right); e and f, multilobed. Scale bar: 10 μm.

**Figure 3:**
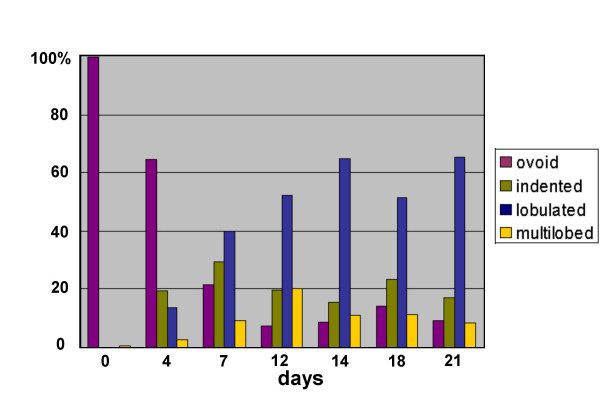
**Time course of nuclear differentiation in RA treated HL-60-*bcl*-2 cells over a three-week period. **The percentage of cells in each of the four nuclear categories from day 0 to day 21 (following addition of RA): ovoid; indented; lobulated; multilobed.

Bcl-2 overexpressing HL-60 cells remain viable for longer periods than the parent cell line, following exposure to RA [18,19]. A significant fraction of RA treated cells survive for up to two weeks; some even for three weeks, exhibiting granulocytic characteristics including: nuclear lobulation, surface antigen expression and nitroblue tetrazolium reduction [18,19]. Figure 2 presents a morphological analysis of Wright-Giemsa stained HL-60-*bcl*-2 cells for up to three weeks following addition of 1 μM RA. For the sake of analysis, we distinguished four nuclear morphology categories: ovoid (Figure 2a), indented (Figure 2b), lobulated (Figures 2c and 2d left) and multilobed (Figures 2d right, 2e and 2f). A multilobed granulocytic nucleus is one that displays five or more lobes, a diagnostic hematological criterion observed in blood smears from humans with megaloblastic anemia [20]. Figure 3 is a graphical representation of nuclear morphological changes during RA induced granulocytic differentiation. At one week following addition of RA, ~80% of the cells reveal nuclear differentiation, with ~95% viability (trypan blue exclusion). From 12 to 21 days post-addition of RA, small changes in the distribution of nuclear morphologies are apparent. Also during this period, cellular debris from dying cells becomes increasingly evident. These observations are in agreement with detailed viability measurements [18], which indicate a significant loss of viability after ~14 days post addition of RA.

**Figure 4:**
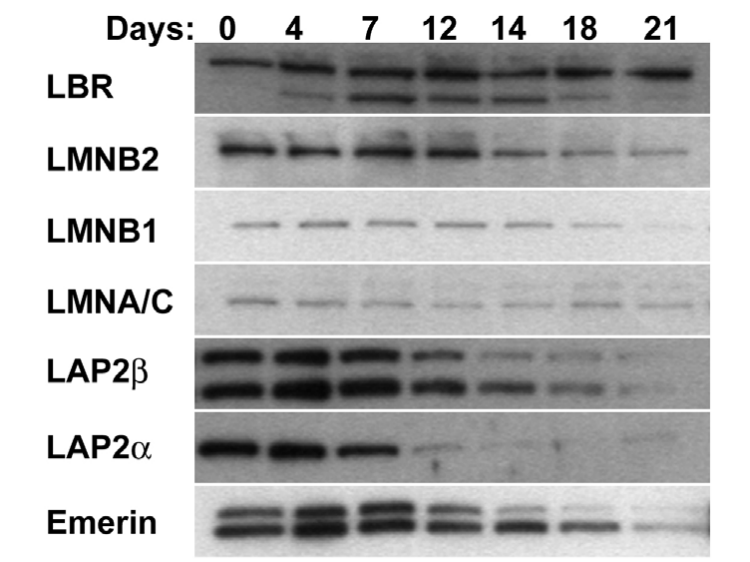
**Immunoblot of total cell extracts of RA treated HL-60-*bcl*-2 cells for up to three-weeks of differentiation. **Nuclear antigens: LBR, lamin B receptor; LMNB2, lamin B2; LMNB1, lamin B1; LMNA/C, lamin A/C; LAP2β; LAP2α; Emerin. The lanes contained comparable amounts of total proteins as judged by Ponceau S staining of the PVDF membrane. Days following addition of RA are indicated.

An immunoblotting analysis from total cell extracts of RA differentiating HL-60-*bcl*-2 cells is presented in Figure 4, focusing upon nuclear envelope components. For a detailed comparison with total cell extracts from differentiating HL-60/S4 cells, see [5]. Based upon densitometric analyses from three separate immunoblotting experiments, LBR showed an increased amount for days 7 to 14 (~2 to 3-fold, compared to the level at day 0). This increase was seen with both the expected ~70 kD protein band and a ~53 kD band, which may represent a proteolytic product [4]. By contrast, other nuclear proteins (lamin B2, LAP2 α, LAP2 β and emerin) exhibited little-or-no increase in levels, but revealed decreases (especially after day 12). LAP2 β and emerin consistently revealed double bands, which might represent protein modification (e.g., phosphorylation). The low levels of lamins A/C and B1 revealed a relative constancy throughout the period of RA-induced granulocytic differentiation, consistent with earlier observations on HL-60/S4 cells [5]. The loss of proteins on (or after) day 12 may reflect a combination of programmed gene expression and programmed protein degradation. Therefore, experiments on the effects of various cytoskeleton-modifying drugs upon HL-60-*bcl*-2 cells were confined to a 10-day period following the addition of RA.

### Confocal immunofluorescence studies of HL-60-*bcl*-2 reveal the existence of micronuclei

**Figure 5:**
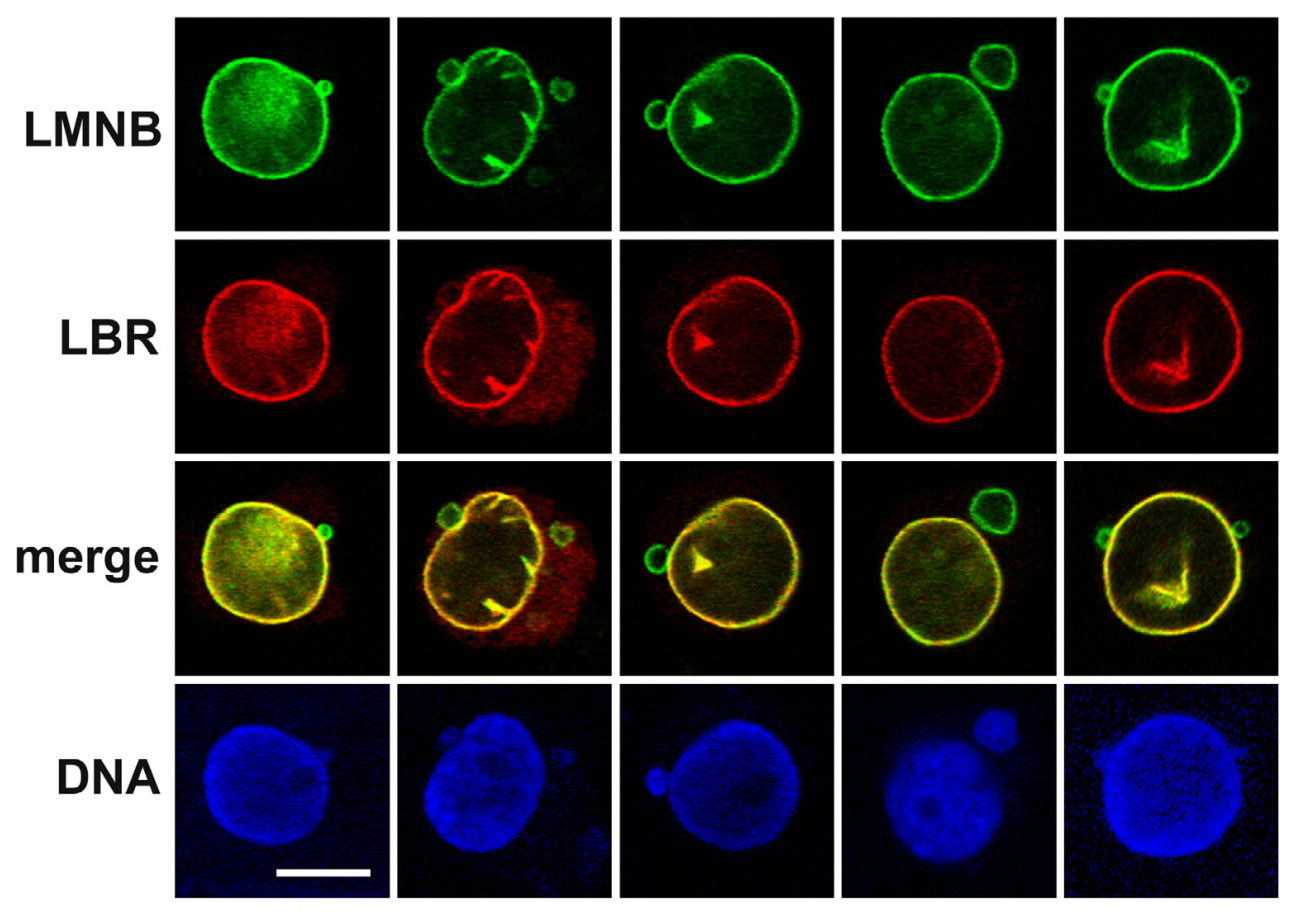
**Gallery of confocal immunofluorescent images of stained nuclei from undifferentiated HL-60-*bcl*-2 cells. **Cells were selected that demonstrate micronuclei adjacent to a main nucleus: LMNB, lamin B; LBR, lamin B receptor; DNA, TO-PRO-3 stain. Notice that micronuclei frequently exhibit a relative deficiency of LBR compared to the main nuclei, but show comparable amounts of LMNB. Scale bar: 10 μm.

**Figure 6:**
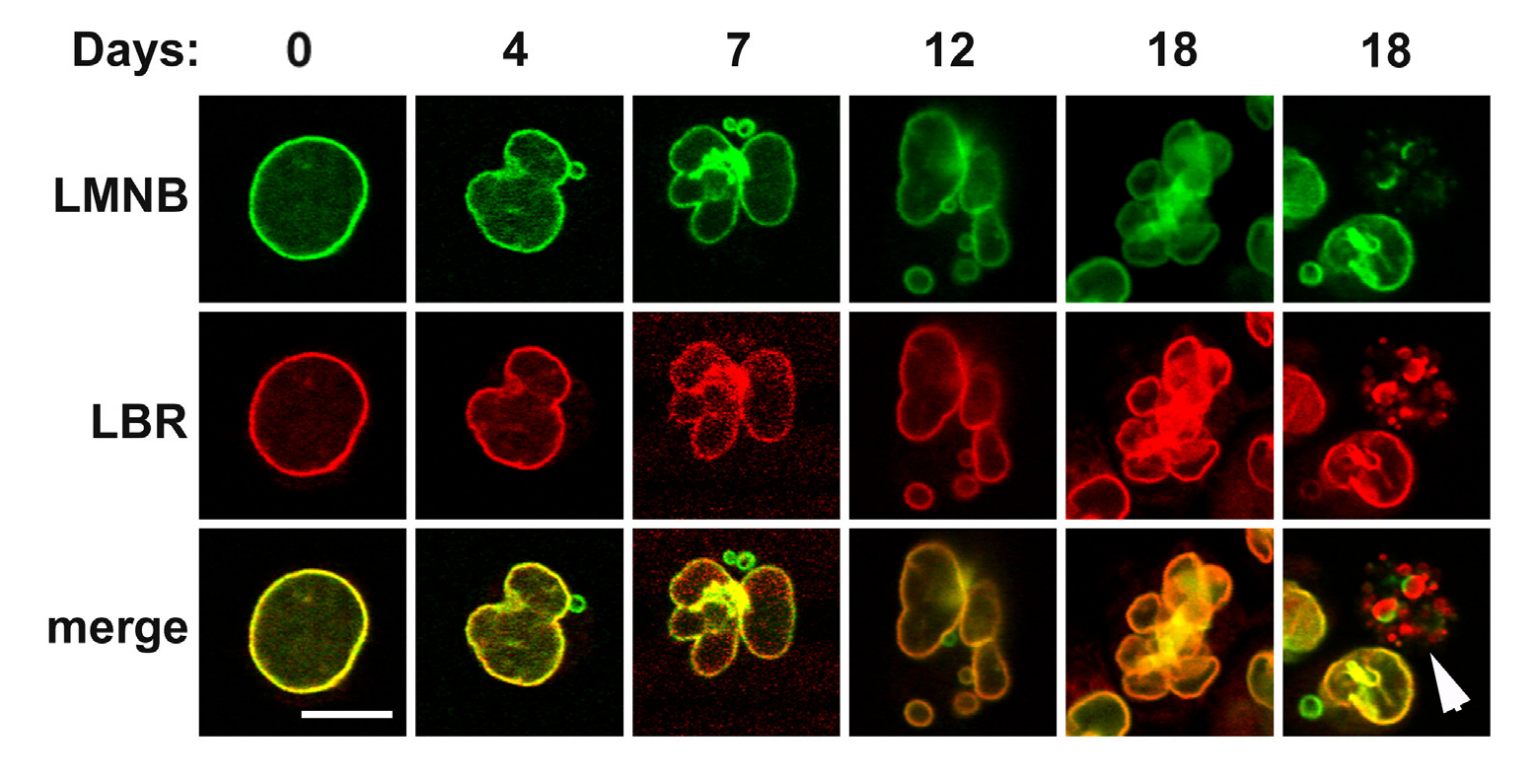
**Gallery of confocal images of LMNB and LBR stained nuclei during RA induced granulocytic differentiation. **Note the increased lobulation and the persistence of micronuclei (frequently exhibiting a relative deficiency of LBR). The arrowhead points to an apoptotic cell with apoptotic bodies. Scale bar: 10 μm.

Immunofluorescent staining was performed on undifferentiated and RA treated HL-60-*bcl*-2 cells to better document nuclear shape and antigen localization. In the course of the experiments on undifferentiated cells, we discovered that ~10% of the cells exhibited one or more micronuclei, in close proximity to the main nucleus. Furthermore, co-localization experiments revealed that the vast majority of micronuclei contained lamin B, but reduced levels of LBR, in comparison to the adjacent main nucleus. Figure 5 presents a gallery of anti-LBR and anti-lamin B stained cells with a single main nucleus and adjacent micronuclei. As revealed by TO-PRO-3 staining, the micronuclei also contained DNA. During granulocytic differentiation the round micronuclei continue to be visualized, even as the main nuclei are undergoing indentation and lobulation (Figure 6). Again the majority of micronuclei maintain a relative deficiency of LBR, compared to lamin B. The nuclear envelope and patches of ELCS (bright yellow regions) appear to possess significant local concentrations of both LBR and lamin B. Also shown in Figure 6 (right column, arrow) is a cell containing apoptotic bodies, exhibiting persistence of LBR staining, with reduced lamin B reactivity, in agreement with observations that lamin B is degraded prior to LBR [21].

**Figure 7:**
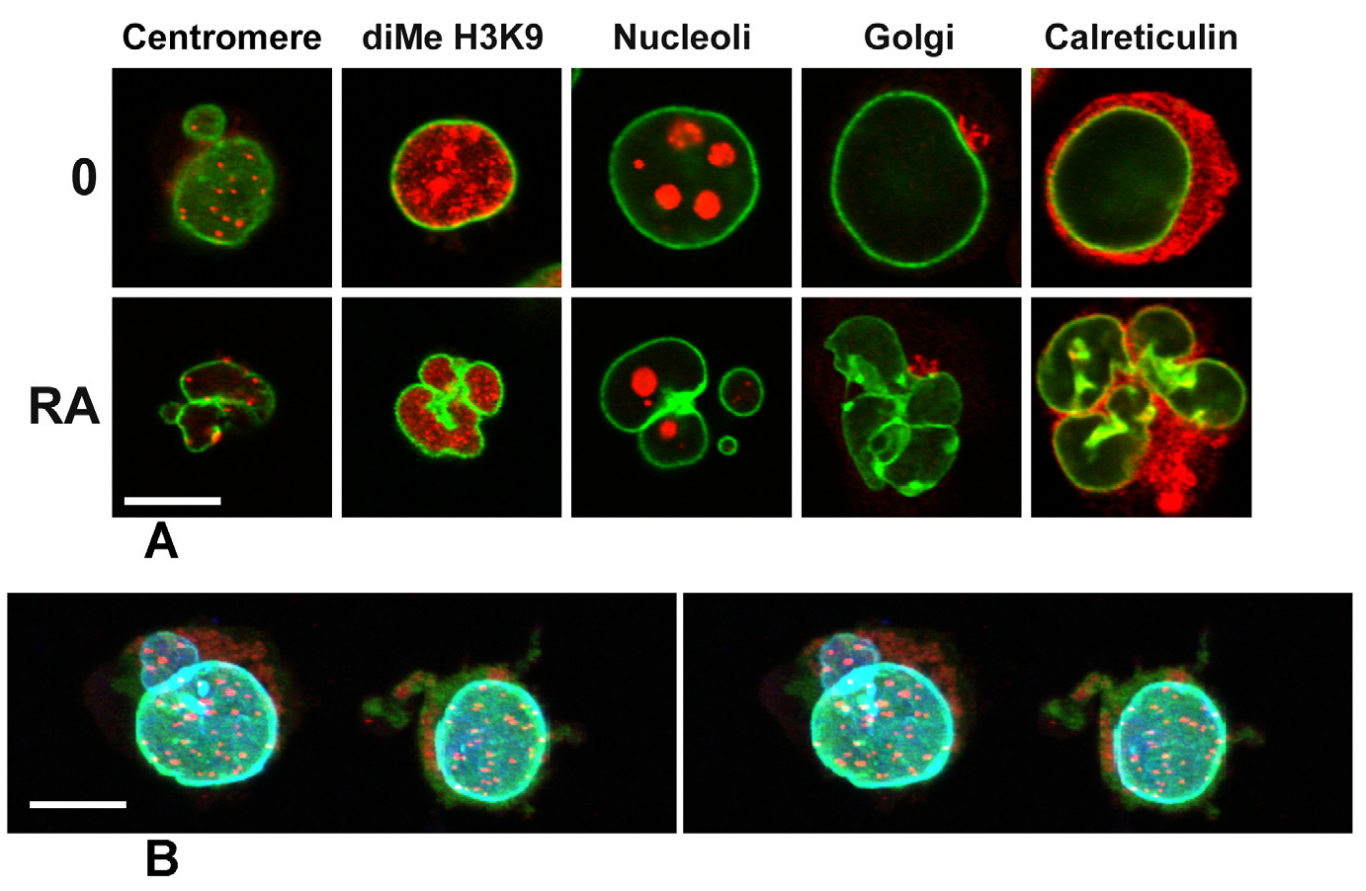
**Confocal immunofluorescent images of HL-60-*bcl*-2 cells. **A. Gallery of undifferentiated and RA treated (7 days) cells. The columns (left to right) are: anti-centromere (CREST); anti-dimethyl H3K9; anti-nucleolus; anti-Golgi p58; anti-calreticulin. LMNB is always shown in green; the different antigens in red. Scale bar: 10 μm. B. Stereo image of undifferentiated HL-60-*bcl*-2 cells stained with anti-centromere (red), anti-LMNB (blue) and anti-LBR (green). Note the presence of centromeres within a micronucleus, which exhibits a deficiency of LBR. Scale bar: 10 μm.

In an effort to better define the nature of the nuclei in differentiating HL-60-*bcl*-2 cells, we explored their immunostaining properties (Figure 7). Our results indicate that main nuclei and many micronuclei contain centromeres (reactivity with CREST antisera), heterochromatin (reactivity with anti-dimethyl H3K9) and nucleolar staining. Figure 7A also indicates that the Golgi apparatus (anti-p58) retains its discrete juxtanuclear form in undifferentiated and granulocytic cell types. In addition, the ER (anti-calreticulin) is present in the spaces between nuclear lobes of granulocytic HL-60-*bcl*-2 cells. A confocal stereo image of immunostained HL-60-*bcl*-2 cells is also presented in Figure 7B, demonstrating a micronucleus deficient in LBR and containing multiple centromeres. Micronuclei have not been reported to occur in granulocytes or during granulopoiesis, underscoring that HL-60 cells are clearly abnormal in certain aspects of nuclear and cellular physiology (see [3] for other differences, compared to normal granulocytes).

### Retinoic acid induced nuclear differentiation of HL-60-*bcl*-2 cells occurs in the presence of cytochalasin D

**Figure 8:**
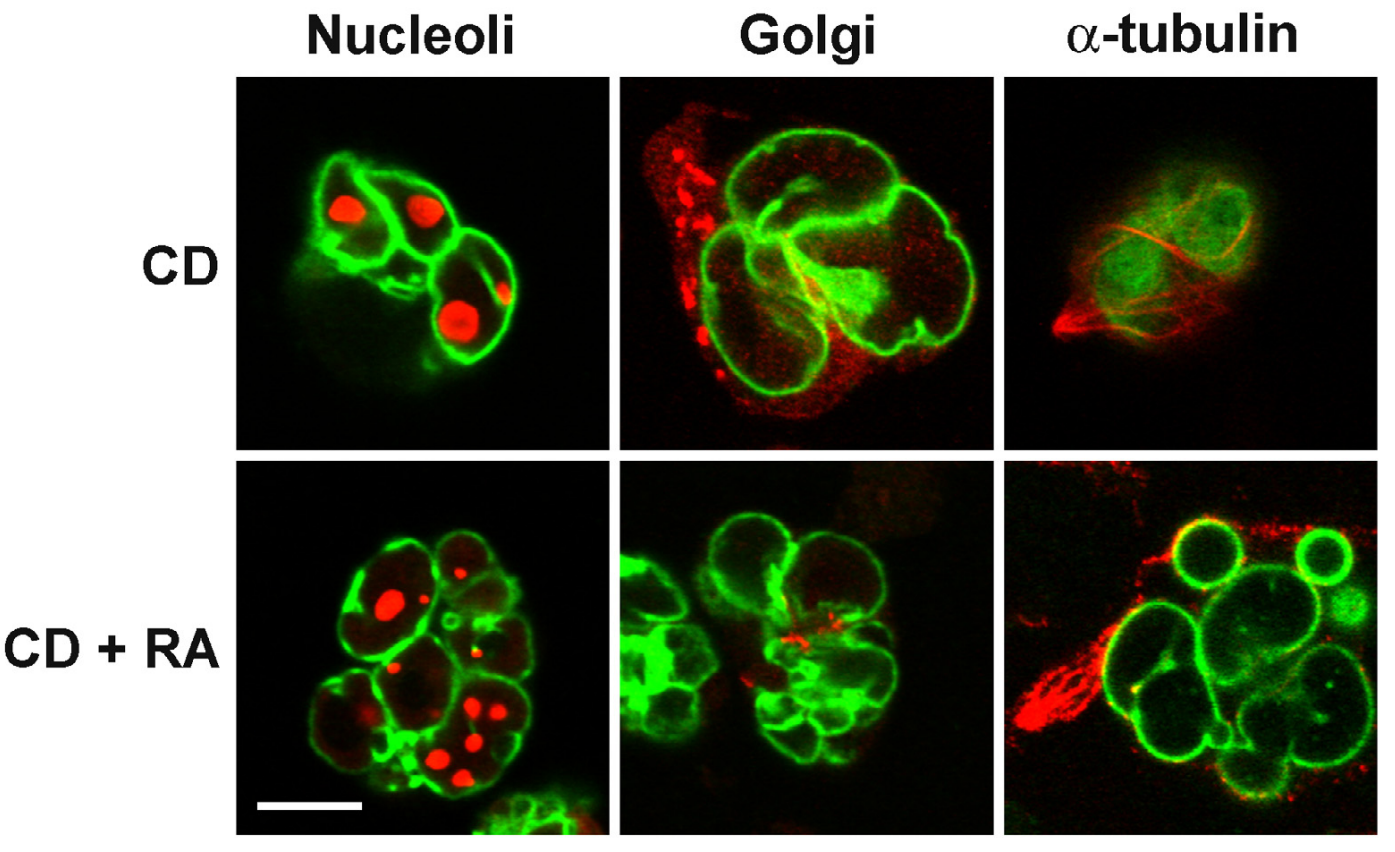
**Gallery of confocal images of CD treated undifferentiated and RA differentiated HL-60-*bcl*-2 cells. **CD and RA treatment were for 7 days. The columns (left to right) are: anti-nucleolus; anti-Golgi p58; anti-α-tubulin. LBR is always shown in green; the other antigens in red.

HL-60*-bcl-*2 were exposed to 1 μM CD with (or without) 1 μM RA for up to 7 days, cytospun onto microscope slides, methanol-fixed, air-dried and stained with Wright-Giemsa. The results were generally similar to those described above for HL-60/S4 cells. Several examples of Wright-Giemsa stained HL-60*-bcl-*2 cells treated with CD or with RA plus CD are shown in Figure 1e,1f,1g,1h. Viability of both the CD-only and the RA plus CD cells were ~60% by day 7. By day 2 following addition of CD with (or without RA), ~70% of the cells were binucleated. A slow progression to tri-, tetra- and >tetranucleated cells was observed, leading to a population with ~20% mono-, ~50% bi- and ~30% multinucleated cells by day 7. Lobulation and ELCS formation was evident in the cells exposed to RA for 7 days; but nuclei within the CD-only cells also revealed distorted and folded nuclear shapes. This was best observed when cytospun HCHO-fixed cells were immunostained and viewed by confocal microscopy (Figure 8). Therefore, it seems reasonable to conclude that CD does not prevent nuclear lobulation and ELCS formation induced by RA. Furthermore, prolonged exposure to CD alone exerts direct (or indirect) effects upon nuclear shape in undifferentiated HL-60*-bcl-*2 cells.

### Nocodazole prevents nuclear lobulation in retinoic acid treated HL-60-*bcl*-2 cells

**Figure 9:**
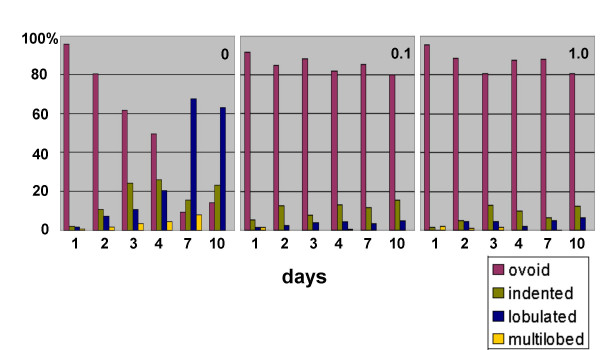
**Nuclear differentiation in RA treated HL-60-*bcl*-2 cells exposed to varying concentrations of NC. **Panels: 0, 0.1 and 1.0 μM NC. The percentage of cells in each of the four nuclear categories from day 0 to day 10 (following addition of RA): ovoid; indented; lobulated; multilobed.

**Figure 10:**
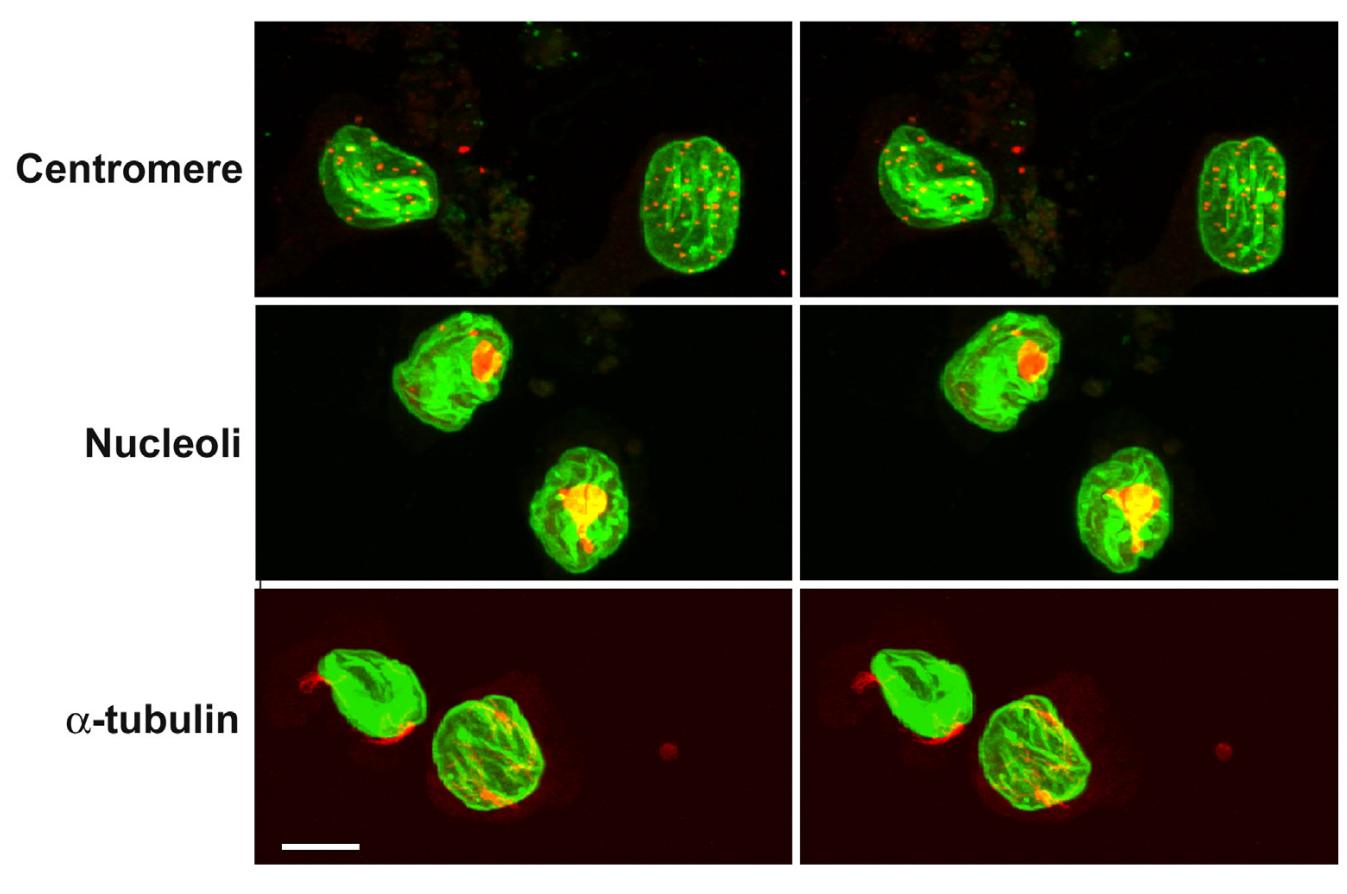
**Stereo confocal images of HL-60-*bcl*-2 cells exposed to 1.0 μM RA and 0.1 μM NC. **Treatment was for 8 days. The rows are: anti-centromere (CREST); anti-nucleolus; anti-α-tubulin. LMNB is always shown in green; the different antigens in red. Scale bar: 10 μm.

**Figure 11:**
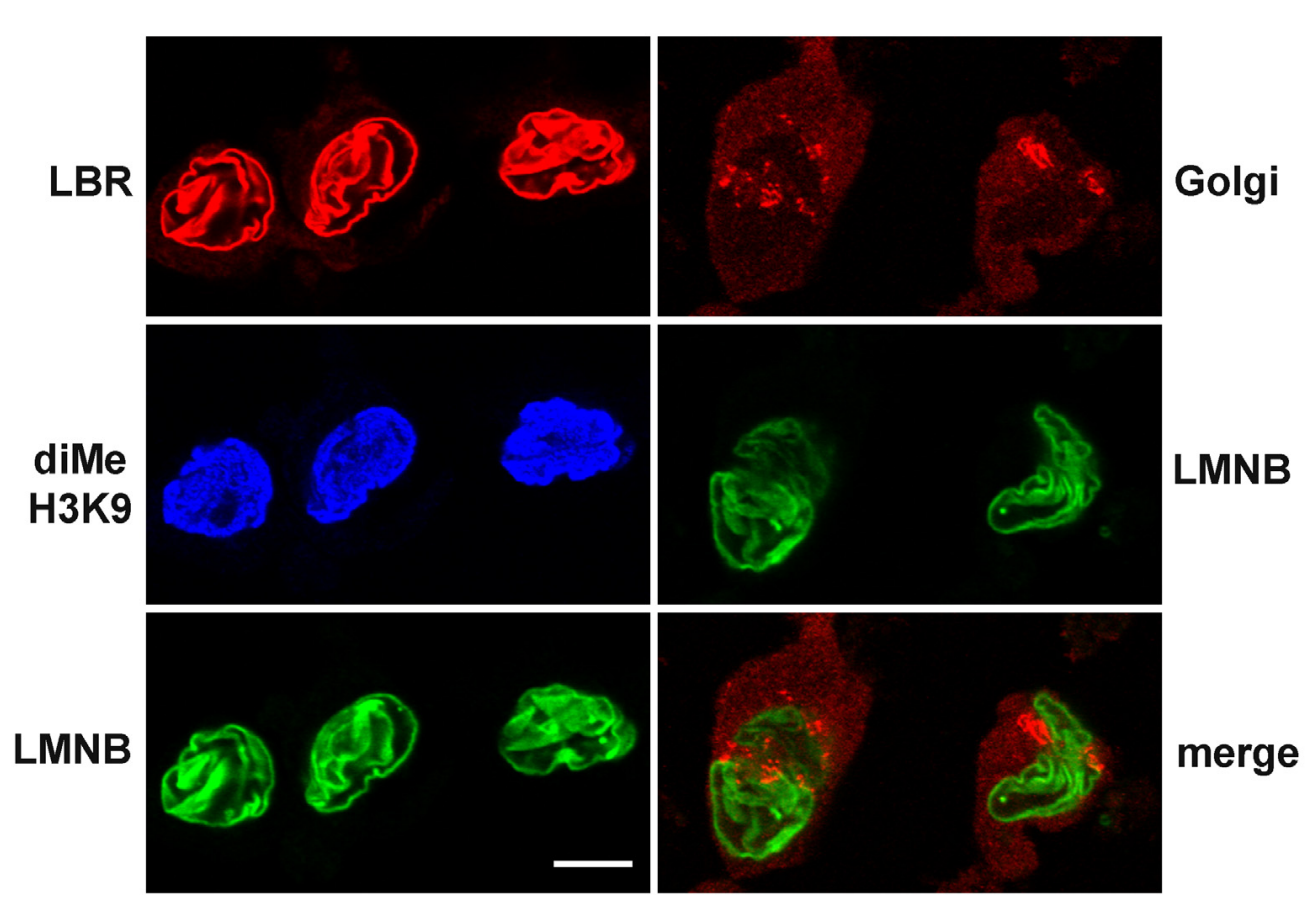
**Confocal images of HL-60-*bcl*-2 cells exposed to 1.0 μM RA and 0.1 μM NC. **Treatment was for 8 days. Left column: top, anti-LBR; middle, anti-dimethyl H3K9; bottom, anti-LMNB. Right column: top, anti-Golgi p58; middle, anti-LMNB; bottom, merge. LMNB is always shown in green. Scale bar: 10 μm.

HL-60*-bcl-*2 cells were exposed to 0, 0.1 or 1.0 μM NC, with 1 μM RA for up to 10 days. Samples were harvested for Wright-Giemsa staining at 1, 2, 3, 4, 7 and 10 days; for immunostaining at day 8. Figure 9 summarizes the distribution of nuclear profiles in the cytospun, methanol-fixed, air-dried and Wright-Giemsa stained preparations. The progressive disappearance of ovoid and increase in indented and lobulated nuclear forms is clear when NC is not present. On the other hand, the presence of 0.1 or 1.0 μM NC blocked the appearance of any significant numbers of indented or lobulated nuclear forms. Ovoid-shaped nuclei were present in at least 80% of the NC-treated cells. Immunostained preparations of 0.1 μM NC treated cells (HCHO-fixed and not air-dried, to preserve 3-D structure) are presented in Figures 10 and 11. The ovoid nuclei give a slightly wrinkled appearance, with considerable surface infolding; but clearly not lobulations. Short tufts of MTs could be visualized in juxtanuclear positions, consistent with a general depolymerization of MTs. The nuclear envelope exhibited clear staining with anti-lamin B and anti-LBR. Nuclear interiors were stained with anti-centromere, anti-nucleolar and anti-dimethylated H3K9 antisera. Anti-Golgi antibodies revealed some dispersion of the p58 antigen. Dispersal of the Golgi resulting from MT disruption is well documented [22]. In one set of experiments (data not shown), NC was added to HL-60-*bcl*-2 cells at day 2 or day 4 after the addition of RA and nuclear morphology was observed on day 8. NC had a clear inhibitory effect upon RA induced nuclear lobulation, even when added 4 days after RA. Thus the inhibitory effect of NC is not an early event in granulocytic differentiation. As noted earlier, we have observed essentially 100% cell death of HL-60/S4 cells exposed to 0.1 μM NC by day 2. Viability of HL-60*-bcl-*2 cells was much better; but protection was not complete. By day 8 of 0.1 μM NC, viability had dropped to ~23%. This could also be observed as increased levels of cellular debris in the Wright-Giemsa stained slides. The cells shown in Figures 10 and 11 were judged to have been viable prior to fixation and staining, based upon the intactness of their nuclear envelopes. Prolonged exposure of cells to NC has clear detrimental effects. But within this limitation, it can be concluded that exposure to NC prevents RA induced nuclear lobulation in HL-60*-bcl-*2 cells.

### Taxol treatment of HL-60-*bcl*-2 cells results in nuclear lobulation and micronuclei, independently of exposure to retinoic acid

**Figure 12:**
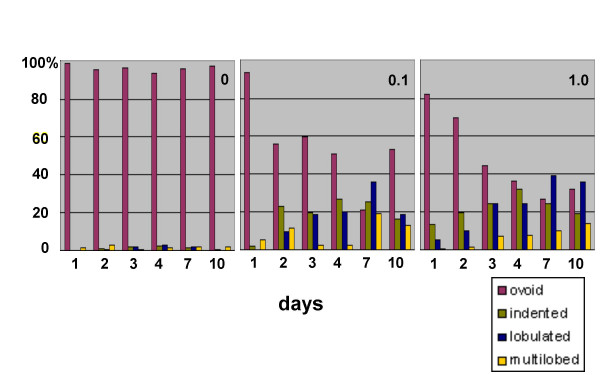
**Nuclear shape changes in undifferentiated HL-60-*bcl*-2 cells exposed to varying concentrations of TX. **Panels: 0, 0.1 and 1.0 μM TX. The percentage of cells in each of the four nuclear categories from day 0 to day 10: ovoid; indented; lobulated; multilobed.

**Figure 13:**
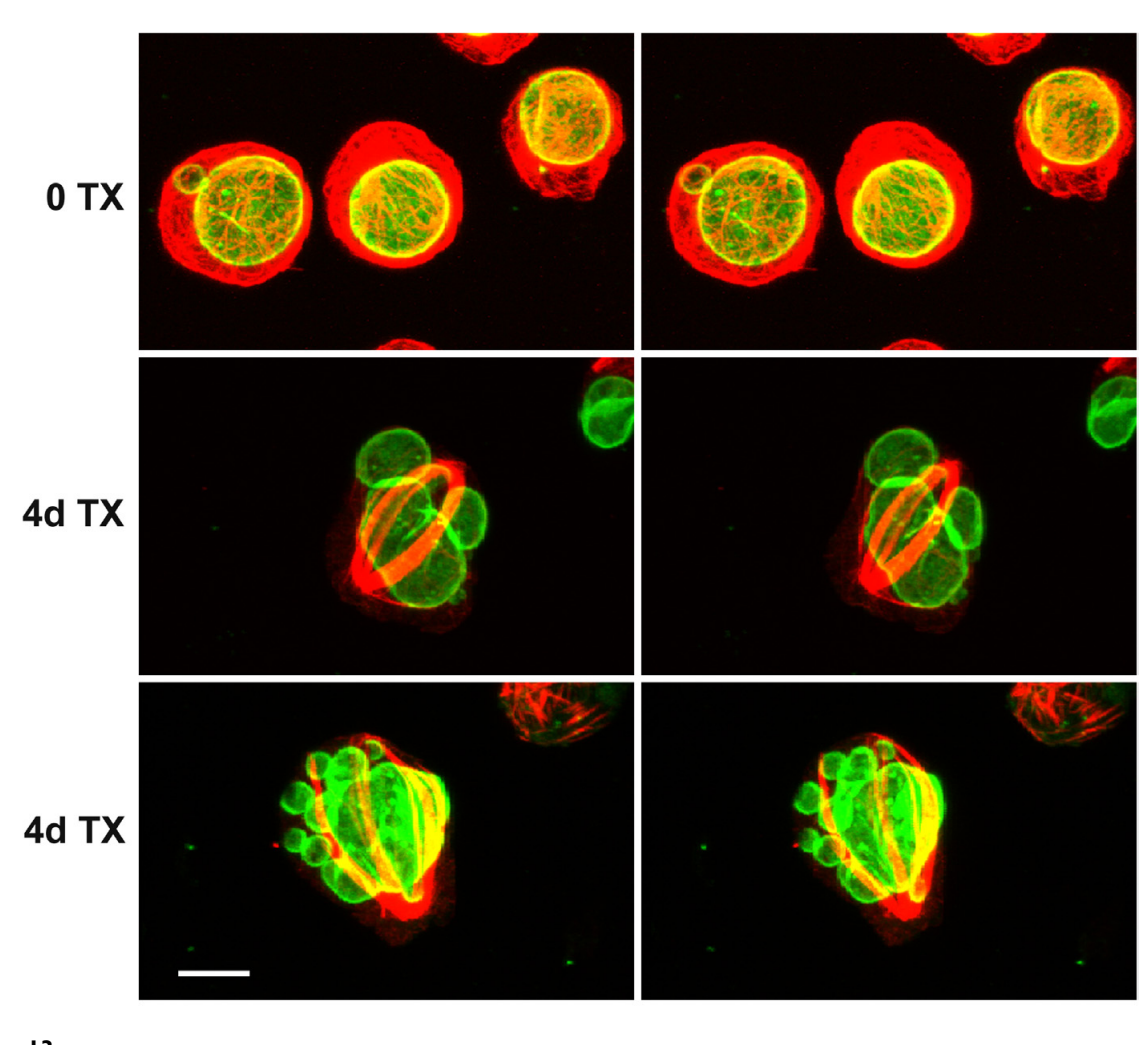
**Stereo confocal images of undifferentiated HL-60-*bcl*-2 cells exposed to 1.0 μM TX. **Treatment was for 4 days. Rows: top, control cells, unexposed to TX; middle and bottom, 4 days of TX. LMNB is shown in green; α-tubulin in red. Scale bar: 10 μm.

**Figure 14:**
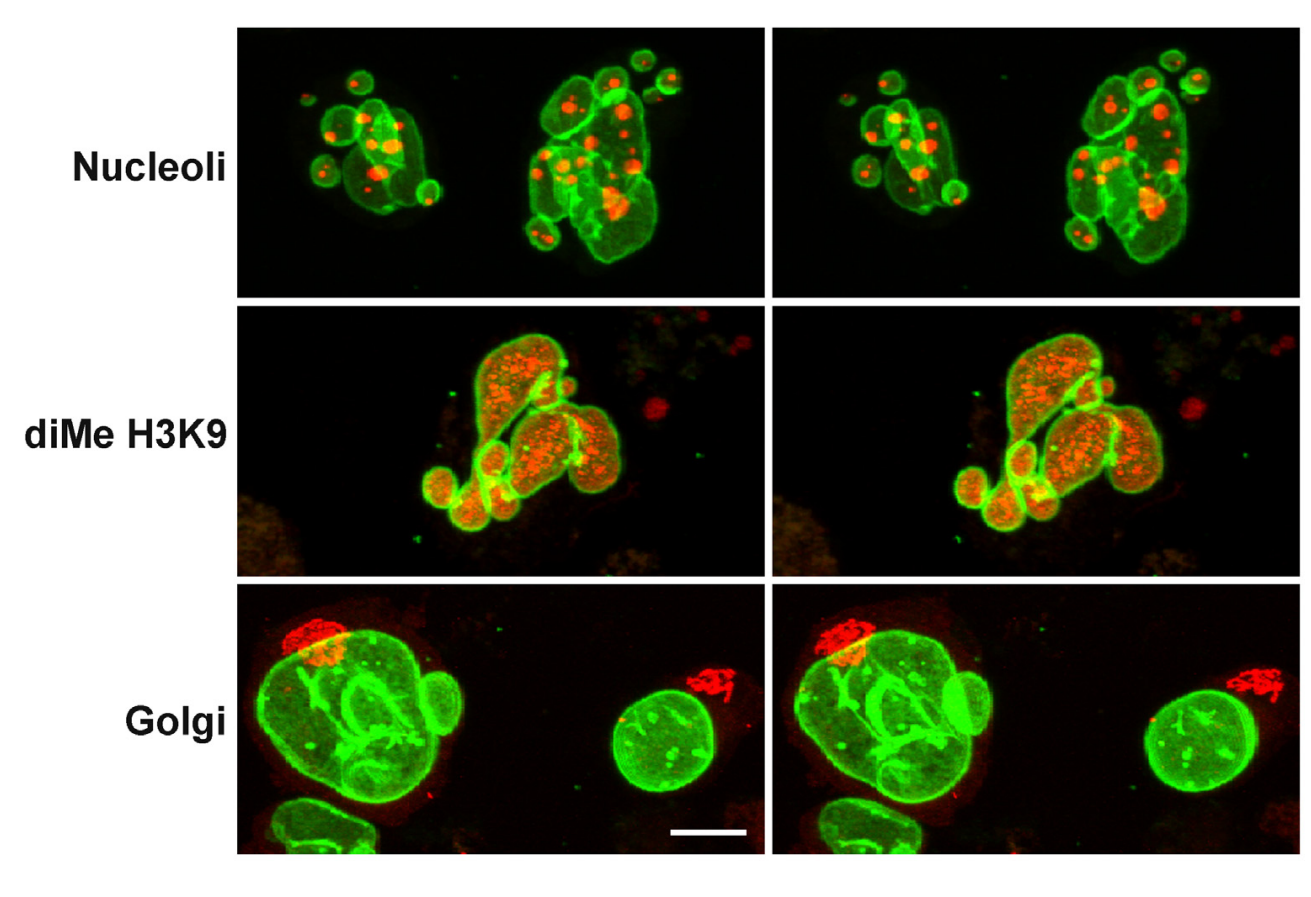
**Stereo confocal images of undifferentiated HL-60-*bcl*-2 cells exposed to 1.0 μM TX. **Treatment was for 4 days. Rows: top, anti-nucleolus; middle, anti-dimethyl H3K9; bottom, anti-Golgi p58. LMNB is shown in green; other antigens in red. Scale bar: 10 μm.

As described earlier, addition of 0.1 μM TX to HL-60/S4 cells resulted in ~100% cell death by day 2, independently of the presence (or absence) of RA. Apparently the cytotoxicity of TX is potentiated in cells with elevated levels of c-*myc *[23]. HL-60 cells are well known to possess c-*myc *amplification [24,25]. Preliminary studies with HL-60-*bcl*-2 cells indicated that as early as day 2, addition of 0.1 or 1.0 μM TX resulted in the appearance of indented and lobulated nuclei in the absence of RA. Consequently, Wright-Giemsa staining analysis of TX treated cells (in the absence of RA) was performed for up to 10 days (Figure 12; identical results were obtained in the presence of RA). There was a rapid decline of cells with ovoid nuclei and a corresponding increase of cells with indented and lobulated nuclear forms. There was also a progressive increase in cell death and debris in the stained preparations. Indeed by day 4, HL-60-*bcl*-2 cells exposed to 0.1 μM TX displayed only ~20% viability. Even so, there were a sufficient number of viable cells to perform immunofluorescent staining. Besides the evident nuclear lobulation, considerable numbers of cells revealed formation of micronuclei. This, combined with the extensive bundling of MTs, resulted in dramatic images of highly perturbed cells (Figure 13). There was no obvious spatial relationship between the positions of the MT bundles and the nuclear lobulations and micronuclei. This may signify that the action of TX on cells occurs earlier (e.g., disruption of the mitotic spindle), with later rearrangements during interphase. TX induced micronuclei revealed heterochromatin (anti-dimethylH3K9) and nucleolar materials (Figure 14). Clearly, the dramatic effects of TX upon nuclear shape do not depend upon the presence of RA. It remains to be demonstrated whether these effects occur by perturbation of interphase and/or mitotic MTs.

### The centrosomal region retains its proximity to the nucleus in HL-60-*bcl*-2 cells under all conditions

**Figure 15:**
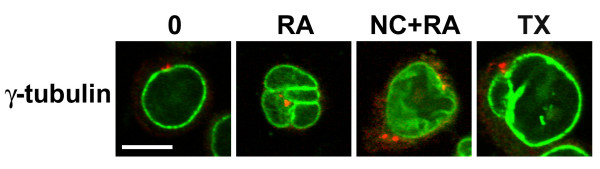
**Confocal images of HL-60-*bcl*-2 cells immunostained for γ-tubulin. **Cell treatments: 0, undifferentiated; RA, 7 days; RA + NC, 7 days with 1 μM RA and 0.1 μM NC; TX, 3 days with 1 μM TX. γ-tubulin is shown in red; LBR in green. Scale bar: 10 μm.

The position of the centrosomal region was visualized with monoclonal antibodies against γ-tubulin (Figure 15). In all the conditions tested (undifferentiated; RA treated; RA and CD treated; TX treated), the centrosomal region appeared to be near the nuclear envelope; often surrounded by nuclear lobes in the RA treated cells. The proximity of the centrosomal region to the nucleus in RA treated HL-60-*bcl*-2 cells contrasts with earlier observations on RA treated "polarized" HL-60/S4 cells [4]. Light microscope observations of living cells revealed fewer polarized HL-60-*bcl*-2 cells, than previously observed for HL-60/S4 cells (data not shown). It is possible that the HL-60/S4 cells are more readily "activated", like normal neutrophils, than are HL-60-*bcl*-2 cells. They also differentiate to granulocytic form faster (~3 to 4 days), compared to HL-60-*bcl*-2 cells (~7 days).

## Discussion

### HL-60-*bcl*-2 cells and granulocytic nuclear differentiation

Prolonged exposure of HL-60 cells to various cytoskeleton-modifying chemicals (i.e., nocodazole and taxol) are very harmful to cell viability, inducing rapid apoptosis. The present study was conducted primarily on a Bcl-2 overexpressing subline of HL-60 cells, which is more refractory to apoptosis and exhibits the chemically induced differentiation properties of the parent cell line [18]. Lethal effects of NC or TX are delayed in HL-60-*bcl*-2 cells, compared to HL-60/S4 cells, allowing a window of time for determining the effects of these MT modifying chemicals on nuclear shape and nuclear differentiation.

There are two additional differences between HL-60-*bcl*-2 and HL-60 cells observed in this study. The first difference: ~10% of the undifferentiated HL-60-*bcl*-2 cells exhibit micronuclei, compared to ~0.8% of HL-60 cells [26]. Micronuclei are generally regarded as the products of abnormal mitoses, where the enclosed chromosomes or chromosome fragments fail to congress at the mitotic plate, but are still surrounded by a post-mitotic nuclear envelope [27-30]. In normal human cells growing in culture (e.g., lymphocytes), they occur in less than 0.5% of the cells [31]. They can be induced in cells by treatment with a variety of DNA breakage conditions (e.g., irradiation) or spindle disrupting agents (e.g., colchicine). Micronuclei in HL-60 cells are described as representing amplified acentric euchromatic genes (such as c*-myc*) and appear to form dynamically during S phase [32]. We observed that micronuclei in HL-60-*bcl*-2 cells exhibited immunostaining of centromeres, heterochromatin and nucleolar antigens (Figure 7), which suggests differences from the earlier interpretation of the nature of micronuclei in HL-60 cells.

Especially puzzling was our observation that the majority of micronuclei possessed comparable amounts of lamin B, but reduced amounts of LBR, in comparison to the companion main nucleus. A recent study employing MCF-7 cells observed micronuclei containing lamins A/C and B1 and a relative deficiency of LBR [33]. The same study demonstrated that when significant numbers of micronuclei were induced by prolonged exposure to the spindle disrupting chemical curcumin, the resulting nuclear envelopes contained LBR. Current views on the sequence of protein additions to post-mitotically reformed nuclei agree that LBR enters the nascent nuclear envelope well before lamin B [34,35]. It is possible that untreated HL-60-*bcl*-2 and MCF-7 micronuclear envelopes form later than the main nucleus, after the cytoplasmic pool of LBR is exhausted, whereas curcumin "induced" micronuclei form at about the same time as main nuclei.

The second difference: RA treated HL-60-*bcl*-2 cells exhibit a small population (up to 20%) with multilobed nuclei, which we almost never observed with HL-60/S4 cells. There may be some clinical significance to this latter observation. A small percent of granulocyte nuclei with 5 or more nuclear lobes in human blood smears is considered diagnostic for megaloblastic anemia (vitamin B12 or folic acid deficiency) [20]. The present data with RA treated HL-60-*bcl*-2 cells suggests that delayed or dysfunctional apoptosis might play a role in these human diseases. It is of interest that neutrophil nuclear multilobulation (hypersegmentation) has been described in two circumstances that delay apoptosis: 1) Glucocorticoid administration to patients induces hypersegmentation [36], and *in vitro *glucocorticoid treatment of neutrophils prolongs their survival [37-39]. 2) Granulocyte colony-stimulating factor (G-CSF) administration to rats induces hypersegmentation in mature neutrophils [40], and *in vitro *treatment of neutrophils with G-CSF prolongs their survival [38,41].

### Major cytoskeletal influences on nuclear shape in HL-60-*bcl*-2

The most important present observation is the suppression of RA induced nuclear lobulation in HL-60-*bcl*-2 cells by simultaneous exposure to NC (Figure 9). This observation implies that MTs must be intact during nuclear differentiation. Furthermore, we have observed (data not shown) that HL-60-*bcl*-2 cells can be made 0.1 μM NC on day 2 or 4 after addition of RA, still exhibiting inhibition of nuclear lobulation. This observation suggests that the requirement for intact MTs is not an early event in the nuclear differentiation process. When RA and NC treated HL-60-*bcl*-2 cells were examined by confocal immunofluorescent staining with anti-lamin B, the ovoid nuclei revealed extensive "wrinkling" of the nuclear envelope (Figures 10 and 11). We suggest that this "wrinkling" reflects expansion (growth) of the nuclear envelope in the absence of nuclear lobulation.

The present study demonstrates a lack of requirement of an intact actin microfilament system for RA induced granulocytic nuclear differentiation (Figure 1). This conclusion is based upon the observation that incubation of HL-60/S4 and HL-60-*bcl*-2 cells with 1 μM CD in the presence of RA does not inhibit nuclear lobulation or the formation of ELCS. Our results agree with an earlier study [17]. In addition, we extended the incubation (with CD and RA) to times where nuclear differentiation is more definitive. Our data also demonstrated that prolonged incubation with CD alone produces significant nuclear envelope folding, which is best appreciated by confocal immunostaining of the nuclei (Figure 8). The mechanism for the nuclear shape changes in undifferentiated cell nuclei exposed to prolonged incubation with CD is presently unknown. There is increasing evidence that actin microfilament interactions with the nuclear envelope exist, mediated via actin binding spectrin-like proteins, variously named Syne 1 and 2/ nesprin-1 and 2/ ANC1/ NUANCE [12-15]. However, a counter argument for the relevance of this actin interacting system to nuclear differentiation in the HL-60 cell system can be made. Nesprin-1 binds directly to lamin A and emerin [14]; but undifferentiated and granulocytic HL-60 cells possess negligible amounts of lamin A, with emerin primarily localized in the cytoplasm [5]. Furthermore, published [13] and unpublished data (A. Karakesisoglu, A. Olins and D. Olins) indicate that HL-60 cells, undifferentiated or RA treated, possess only trace amounts of NUANCE.

The finding that TX treatment of cells has dramatic consequences to interphase nuclear structure has been reported before. Exposure of human carcinoma cells (Ishikawa and HeLa) to 0.01–0.1 μM TX for up 20 hours, followed by incubation in drug-free media for up to 72 hours, led to nuclear envelope "unraveling" and clustering of nuclear pores [42]. The authors observed lobulated and micronuclei, much as observed here. In the case of HL-60-*bcl*-2, the nuclear structural changes superficially mimic the effects of RA treatment, but occur much faster (Figure 12). The bundling of MTs in HL-60 cells in response to exposure to 1.0 μM TX for 24 hours has also been reported [43], but no mention was made of the nuclear envelope changes. Furthermore, the authors noted that at 0.1 μM TX (or greater) the HL-60 cells showed clear apoptosis by 24 hours. In a later paper by the same group [44], increased expression of Bcl-2 or Bcl-x_L _yielded HL-60 cells with considerably greater resistance to TX induced apoptosis. It seems to us that the dramatic cell and nuclear changes that we observe have no obvious relationship to normal granulocytic nuclear lobulation, but underscore an effect of MT integrity upon nuclear shape. The formation of micronuclei, in particular, suggests that TX may exert its effects on nuclear shape by interfering with normal mitotic chromosome distribution.

### A model for granulocytic nuclear lobulation

**Figure 16:**
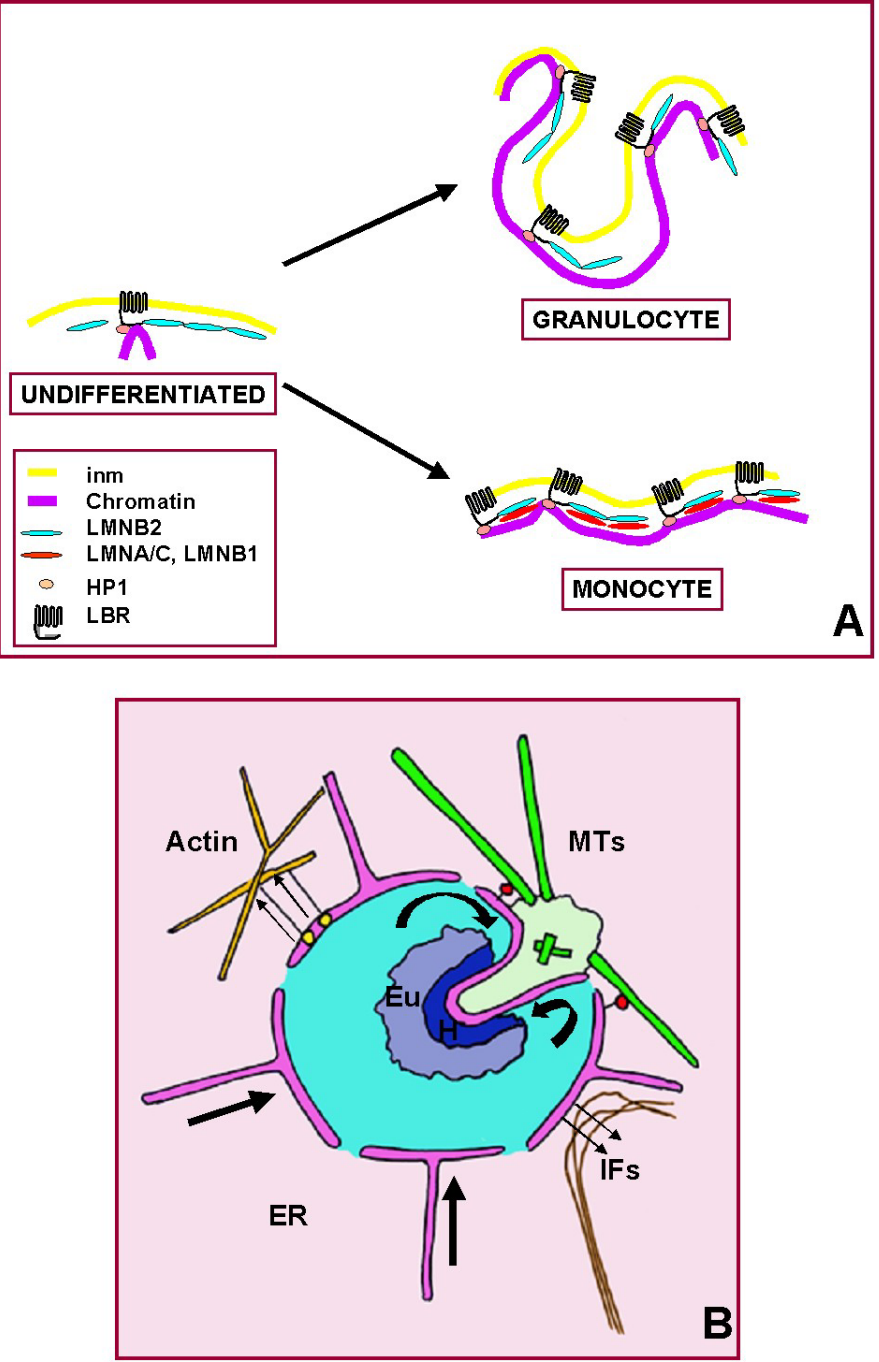
**Model for granulocytic nuclear lobulation. **A. Postulated changes in nuclear envelope flexibility arising from changes in nuclear envelope composition. HL-60 cell states: undifferentiated; granulocyte, RA treated; monocyte, TPA treated. Abbreviations: inm, inner nuclear membrane; LMNB2, lamin B2; LMNA/C, lamins A/C; LMNB1, lamin B1; HP1, heterochromatin protein 1; LBR, lamin B receptor. Due to a paucity of lamins A/C and B1, the nuclear envelope is believed to be more flexible in the undifferentiated and granulocytic cell states. B. Balance of forces postulated to be affecting granulocytic nuclear shape. Microtubules (green) and affiliated dynein motors (red circles) are assumed to produce nuclear envelope invaginations (bent arrows). Actin with affiliated spectrin-like proteins and vimentin are assumed to be pulling outwards on the nuclear envelope (thin arrows). Current evidence does not favor a major contribution by actin; any significant contribution by vimentin is presently unknown. The large straight arrows indicate continued influx of nuclear envelope components from the endoplasmic reticulum, allowing sustained membrane growth. Abbreviations: MTs, microtubules; IFs, intermediate filaments; ER, endoplasmic reticulum; Eu, euchromatin; H, heterochromatin. The nuclear compartment is colored blue; the cytoplasm, pink.

Although far from a complete understanding of all the molecular forces involved in shaping the granulocytic nucleus, a working model that incorporates existing data and emerging concepts provides a useful perspective for future experimentation (Figure 16). The process of granulocytic nuclear lobulation can be viewed as a dynamic balance of stabilizing and distorting forces. The working model contains the following assumptions: 1) the flexible nuclear envelope (due to the paucity of lamins A/C and B1) is "tacked down" to the underlying heterochromatin (enhanced by the elevated LBR); 2) the nuclear envelope undergoes invaginations in the region of the centrosome due to dynein movement along MTs; 3) new membrane materials are added to the nuclear envelope via lateral diffusion from the ER, resulting in net membrane growth [3]; 4) constraints on nuclear shape by actin and spectrin-like proteins are weak, due to the paucity of lamins A/C and NUANCE and the cytoplasmic localization of emerin; 5) constraints on nuclear shape by vimentin-envelope interactions are minimal in the differentiating HL-60 cell system.

We suggest that nuclear envelope deformability is an important factor and depends upon the amount of underlying lamins: the less lamin protein, the more pliable the nuclear envelope. Granulocytic forms of HL-60 exhibit deficiency of lamins A/C and B1, whereas both types of lamins are present in monocyte/macrophage forms [5]. The absence of lamins A/C in normal granulocytes and their presence in macrophages has been previously noted [45]. The present data on granulocytic differentiation in HL-60-*bcl*-2 demonstrates that nuclear lobulation correlates with low levels of lamins A/C and B1 coupled with a rise in LBR levels (Figure 4).

The pivotal role of LBR in determining granulocytic nuclear lobulation was demonstrated with the human Pelger-Huet anomaly and murine Ichthyosis mutations [8,9]. These studies demonstrated that LBR functions in a dose-dependent manner: homozygous mutants present a more severe phenotype and lower amounts of LBR, than in the heterozygous state. The influence of LBR on granulocytic nuclear lobulation is consistent with its known properties [46]. LBR is embedded within the nuclear envelope inner membrane via 8 transmembrane domains (the C-terminal ~400 aa), and associated with lamin B, chromatin and HP1α in the N-terminus (~200 aa). In the absence of sufficient LBR, nuclear lobulation is prevented and the normally peripheral heterochromatin is redistributed into a more centrally condensed form [8,9].

A role for MT integrity is implicit in our present observation that exposure of HL-60-*bcl*-2 cells to NC prevents nuclear lobulation during exposure of the cells to RA. Direct interaction between MTs and the interphase nuclear envelope in mammalian cells have not documented. However, a recent model for mitotic nuclear envelope breakdown [47,48] can be adapted to the situation of nuclear lobulation. The nuclear envelope breakdown model proposes that cytoplasmic dynein attaches MTs to the nuclear envelope, pulling the envelope towards the centrosomal region. The excess envelope near to the centrosome produces nuclear invaginations; the tension on the non-growing nuclear envelope generates tears and the mixing of nuclear and cytoplasmic materials. If we assume that the nuclear envelope is still growing in the case of RA differentiating HL-60 cells, invaginations and lobulations might be expected to accumulate within the intact nuclear envelope. The present study demonstrates proximity of the centrosome to nuclear lobulation (Figure 15). But as yet, there is no direct evidence for cytoplasmic dynein playing a role in granulocytic nuclear differentiation.

Our present data suggests that an intact actin microfilament system does not play a major role in granulocytic nuclear lobulation (Figure 1). The best described mechanism of actin interacting with the nuclear envelope involves spectrin-like proteins, which may bridge cytoplasmic actin to nuclear envelope proteins, such as lamin A and emerin [12-15]. But undifferentiated and granulocytic HL-60 cells possess very little lamin A/C and emerin is primarily cytoplasmic [5], suggesting that this bridging system may not be functional in these cell forms.

In SW-13 cells the absence of intermediate filaments (vimentin) has been correlated with nuclear envelope folds or invaginations [16]. We have observed a decrease in vimentin during differentiation of granulocytic HL-60/S4 cells [3-5], suggesting that reduced vimentin concentrations may contribute to granulocytic nuclear lobulation.

The proposed model for granulocytic nuclear lobulation yields several testable predictions: 1) Expression of lamins A/C and B1 in HL-60 cells should strengthen the nuclear envelope, minimizing nuclear lobulation following exposure of the cells to RA; 2) "Knock-down" or expression of a "dominant negative" form of LBR in HL-60 cells would be expected to suppress granulocytic lobulation; 3) Overexpression of dynamitin in HL-60 cells should inhibit dynein activity [49], preventing nuclear lobulation following RA induced differentiation. A number of these experiments are already in progress.

## Conclusions

Employing Bcl-2 overexpressing HL-60 cells, which are more refractory to induced apoptosis than the parent cell line, we demonstrated that disruption of the MTs by nocodazole prevented nuclear lobulation in response to RA treatment. These results implicate the necessity of an intact MT system for granulocytic nuclear shape. Cytochalasin D, on the other hand, did not suppress RA induced nuclear lobulation. Combined with the decreasing levels of intermediate filaments (vimentin) during differentiating granulocytic forms of HL-60 cells, the role of the MT system appears to be quite central to the nuclear shape change. Recent models on the role of a MT bound motor (dynein) in facilitating mitotic nuclear envelope breakdown suggest that similar tension forces on the differentiating granulocyte nucleus, combined with continued influx of nuclear envelope components, could explain nuclear invaginations and lobulation.

## Methods

### Cells and chemicals

Two HL-60 cell sublines were employed in this study: HL-60/S4 [50], which achieves maximum nuclear lobulation in 4 days following addition of RA; HL-60-*bcl*-2 [18], which achieves maximum lobulation in ~7 days, comparable to the parent cell line. Cultivation conditions were exactly as described previously [3,4].

The following chemicals were purchased from Sigma-Aldrich (St. Louis, MO): *all-trans *retinoic acid (RA), nocodazole (NC), taxol (TX), cytochalasin D (CD). Stock solutions of these chemicals were stored at -20°C as described earlier [3,4].

### Antibodies

Guinea pig antisera were the generous gifts of two Ph. D. students in the laboratory of H. Herrmann (German Cancer Research Center, Heidelberg): anti-LBR and anti-emerin, from C. Dreger; anti-lamin A and anti-lamin B1, from J. Schumacher. Goat anti-lamin B was obtained from Santa Cruz Biotechnology Inc. (Santa Cruz, CA). Rabbit anti-4 × dimethyl H3K9 (lysine 9 on histone 3) was generously provided by T. Jenuwein (Biocenter, Vienna). Human auto-antisera anti-centromere (CREST) and anti-nucleolus (antigens unknown) were purchased from Antibodies Inc. (Davis, CA). Rabbit anti-calreticulin was from Calbiochem (San Diego, CA). Mouse monoclonal antibodies against α-tubulin, γ-tubulin and Golgi p58 were all purchased from Sigma-Aldrich. Mouse monoclonal anti-vimentin (3B4) was a gift of H. Herrmann and has been described before [51]. FITC-, Cy3-, Cy5- and HRP-conjugated donkey secondary antibodies were all purchased from Jackson ImmunoResearch Laboratory, Inc. (West Grove, PA). TO-PRO-3 and SlowFade were obtained from Molecular Probes, Inc. (Eugene, OR).

### Fixation and staining

For Wright-Giemsa staining, cells were cytospun onto ethanol-cleaned microscope slides, fixed in room temperature methanol for 15 min, air-dried and stained as described earlier [3]. For analysis of the percentage of cells in the various nuclear morphology categories (Figures 3, 9 and 12), approximately 150 cells were observed and classified in each experiment.

In the majority of immunostaining experiments the procedure followed that described previously [4], with some modifications, as follows: 1) Microscope slides were soaked overnight in 1/1 ethanol/ether and freshly coated with poly-L-lysine (MW ~150–300,000; Sigma-Aldrich), just before centrifugation of the cells. 2) No coverslip was used during antibody incubations, to minimize loss of cells. 3) Prior to the application of primary antibodies, slides were incubated with 5% normal donkey serum (Jackson ImmunoResearch Laboratory) in PBS for 15–30 min at 37°C in a moist chamber. 4) In the cases of monoclonal anti-γ-tubulin and anti-vimentin, where antigenicity appeared to be destroyed by HCHO fixation, slides were fixed in methanol (-20°C, 10 min), acetone (-20°C, 1 min) followed by three washes in PBS (5 min each). The cells were not excessively flattened by either procedure, maintaining sufficient 3-D structure to justify stereo viewing. Confocal images were collected on a Zeiss 510 Meta. Stereo images were obtained as ± 15° projections through the stack of confocal images.

### Immunoblot analysis

SDS total cell extracts, obtained from undifferentiated and RA treated HL-60-*bcl*-2 cells over a three-week period (simultaneously with preparation of slides for Wright-Giemsa staining), were analyzed by immunoblotting as previously described [5]. No attempt was made to separate viable cells from debris prior to SDS extraction. Comparable amounts of total cell protein were loaded into each lane of the SDS-PAGE, as judged by Ponceau S staining of the PVDF membrane after protein transfer. Several different ECL exposures were collected on X-ray film and subsequently scanned with a Bio Rad Chemi Doc for densitometric analyses.

## Abbreviations

RA, retinoic acid; MT, microtubules; NC, nocodazole; TX, taxol; CD, cytochalasin D; ELCS, nuclear envelope-limited chromatin sheets

## Authors' contributions

ALO performed the microscopy and prepared the figures. DEO performed the tissue culture, immunostaining and immunoblotting. Both authors were involved in the conception of the study and have read and approved the final manuscript.
